# Functional and prognostic significance of long non-coding RNA MALAT1 as a metastasis driver in ER negative lymph node negative breast cancer

**DOI:** 10.18632/oncotarget.9622

**Published:** 2016-05-26

**Authors:** Mahdieh Jadaliha, Xinying Zong, Pushkar Malakar, Tania Ray, Deepak K. Singh, Susan M. Freier, Tor Jensen, Supriya G. Prasanth, Rotem Karni, Partha S. Ray, Kannanganattu V. Prasanth

**Affiliations:** ^1^ Department of Cell and Developmental Biology, University of Illinois, Urbana, IL, USA; ^2^ Department of Biochemistry and Molecular Biology, The Hebrew University-Hadassah Medical School, Jerusalem, Israel; ^3^ Onconostic Technologies, Champaign, IL, USA; ^4^ Ionis Pharmaceuticals Inc., Carlsbad, CA, USA; ^5^ Interdisciplinary Health Sciences Initiative, University of Illinois, Urbana, IL, USA; ^6^ Department of Surgery, University of Illinois College of Medicine, Champaign, IL, USA; ^7^ Carle Cancer Center, Urbana, IL, USA

**Keywords:** breast cancer, nuclear speckle, triple negative breast cancer, lncRNA, basal-like breast cancer cells

## Abstract

MALAT1 (metastasis associated lung adenocarcinoma transcript1) is a conserved long non-coding RNA, known to regulate gene expression by modulating transcription and post-transcriptional pre-mRNA processing of a large number of genes. MALAT1 expression is deregulated in various tumors, including breast cancer. However, the significance of such abnormal expression is yet to be fully understood. In this study, we demonstrate that regulation of aggressive breast cancer cell traits by MALAT1 is not predicted solely based on an elevated expression level but is context specific. By performing loss- and gain-of-function studies, both under in vitro and in vivo conditions, we demonstrate that MALAT1 facilitates cell proliferation, tumor progression and metastasis of triple-negative breast cancer (TNBC) cells despite having a comparatively lower expression level than ER or HER2-positive breast cancer cells. Furthermore, MALAT1 regulates the expression of several cancer metastasis-related genes, but displays molecular subtype specific correlations with such genes. Assessment of the prognostic significance of MALAT1 in human breast cancer (n=1992) revealed elevated MALAT1 expression was associated with decreased disease-specific survival in ER negative, lymph node negative patients of the HER2 and TNBC molecular subtypes. Multivariable analysis confirmed MALAT1 to have independent prognostic significance in the TNBC lymph node negative patient subset (HR=2.64, 95%CI 1.35 − 5.16, p=0.005). We propose that the functional significance of MALAT1 as a metastasis driver and its potential use as a prognostic marker is most promising for those patients diagnosed with ER negative, lymph node negative breast cancer who might otherwise mistakenly be stratified to have low recurrence risk.

## INTRODUCTION

Breast cancer is the leading cause of cancer death in women worldwide [1]. Like many epithelial tumors, breast cancer (BC) is also a heterogeneous disease with multiple subtypes having been elucidated that are substantially different with regard to their clinical phenotypes and therapeutic response profiles. The most commonly employed clinical subtyping system is based on the presence or absence of three receptors: estrogen receptor (ER), progesterone receptor (PR) and human epidermal growth factor receptor 2 (HER2). Based on this, breast cancers are classified into main categories: luminal A (ER positive and/or PR positive and HER2 negative), luminal B (ER positive and/or PR positive and HER2 negative or positive), HER2+ (ER and PR negative, HER2 positive) and triple negative (ER/PR/HER2 negative). Triple-negative breast cancers (TNBC) are also heterogeneous, further sub-classified primarily into basal-like and claudin-low molecular subtypes based on gene expression profiling studies [2–7]. Unfortunately, TNBC is associated with poor prognosis and lacks effective targeted therapeutic options like Tamoxifen (Nolvadex™) or Trastuzumab (Herceptin™) that have been quite successful in treating luminal A/B or HER2+ cancers, respectively [8, 9]. There is thus an urgent need to develop prognostic markers that can better stratify TNBC patients with regard to their risk of disease recurrence, as well as therapeutic targets that are specifically tailored to TNBC. A better understanding of the biology of TNBC thus holds promise in our quest for superior therapeutic treatment options for patients diagnosed with TNBC.

Research efforts to elucidate the biology of breast cancer have thus far been focused on the functional characterization of protein-coding genes. Less than 2% of the human genome encodes for protein whereas up to 80% of the genome is transcribed into RNA [10–12]. Thus, the genome contains a huge repertoire of non-coding RNAs (ncRNAs) [10–15]. NcRNAs come in different sizes from microRNAs of ~20-22 nucleotides (nts) to long non-coding RNAs (lncRNAs) of ~200 to thousands nts in length. Among the ncRNA categories, we know the least about the function of lncRNAs [16, 17]. However, a number of recent studies indicate the involvement of lncRNAs in cancer progression, including in breast cancer [18–28]. For example, HOTAIR lncRNA is overexpressed in breast cancer patients and represses the expression of metastasis suppressor genes, thereby promoting metastasis [21]. Another example includes GAS5 lncRNA, a cell cycle regulator, which is down regulated in breast cancer samples [29]. Some of the candidate lncRNAs that are shown to be aberrantly expressed in breast cancer include H19 [26, 30], aHIF [31], BCYRN1 [32], UCA1 [33], SRA RNA [34], ZFAS1 [35], CCAT2 [36], LSINCT5 [37], NKILA [25], treRNA [20], Eleanors [38] and MALAT1 [39–42]. Recent bioinformatics studies have identified several novel lncRNAs, the expression of which is altered in breast cancer patients: some of these have the potential to be used as prognostic markers [43–45]. Besides this, lncRNAs also play vital roles in mammary gland development [46, 47]. These reports indicate that lncRNAs could be influencing vital processes that are required for mammary cell development, and their aberrant expression could contribute to breast tumor growth and metastasis.

MALAT1 is a long (~8kb), highly conserved nuclear-enriched lncRNA, which is abundantly and ubiquitously expressed in different tissues [48]. In the nucleus, MALAT1 is preferentially enriched in nuclear speckles, which is a non-membranous nuclear structure that is known to regulate key post-transcriptional RNA processing, including pre-mRNA splicing and mRNA export [49–52]. Previous studies reveal that MALAT1 modulates alternative splicing of pre-mRNA by regulating the activity of several of the pre-mRNA processing factors, including SR-family of splicing factors [51, 52]. In addition, MALAT1 is involved in transcriptional regulation [53, 54]. This is consistent with two recent reports where MALAT1 interacts with actively transcribed gene loci, and preferentially interacts with genes, the transcripts of which undergo alternative pre-mRNA splicing [55, 56]. At the cellular level, MALAT1 regulates cell proliferation by controlling the transcription and pre-mRNA processing of key cell cycle-regulated genes [52]. Involvement of MALAT1 in cancer metastasis was first reported in the case of non-small cell lung cancer patients, where overexpression of MALAT1 was positively correlated with lung cancer metastasis [49]. Clinically significant upregulation of MALAT1 is observed in several other tumors, including those of the lung, bladder, breast, cervix, colon, prostate, stomach and liver as well as in osteosarcoma [57, 58].

As mentioned earlier, breast cancer (BC) is a heterogeneous disease and is categorized into several subtypes. It is reasonable to believe that the MALAT1 interacting protein partners and its target genes, the activities of which are regulated by MALAT1, are potentially unique for each of the BC subtypes. It is also not clear whether MALAT1 has the potential to drive tumorigenicity in various subtypes of breast cancer. It has recently been reported that treatment of ER-negative basal-like breast cancer cell lines with high concentration of 17b-Estradiol (E2) decreased the cellular levels of MALAT1, with a concomitant decrease in cell proliferation, migration and invasion [42]. These results suggest a positive correlation between the low levels of MALAT1 and reduced invasion and migration in basal-like BC cells. In the present manuscript we investigated the potential involvement of MALAT1 in inducing tumor progression in different subtypes of breast cancers.

Based on data obtained from various BC cell lines and patient samples, we observed elevated levels of MALAT1 in luminal subtype of breast cancers. However, our functional analyses in different BC cell lines illustrate that the role of MALAT1 in tumor progression is not limited to luminal subtype; rather MALAT1 is also actively involved in the tumor progression in other BC subtypes, including TNBC cells. Furthermore, the disease-specific survival data in patients indicated that MALAT1 level could be used as a potential prognostic marker in the case of TNBC and HER2+, but not luminal patients. More accurately, MALAT1 level in TNBC and HER2+ subtypes could be quantified to predict tumor recurrence and metastasis in lymph-node negative (LN-) patients. Thus, our results support the potential use of monitoring MALAT1 level as a prognostic predictor of tumor recurrence and metastasis in patients diagnosed with ER negative lymph node negative BC. This would potentially help reduce the occurrence of both under-treatment of those hitherto mistakenly assigned to have a low disease recurrence risk, as well as over-treatment of those mistakenly assigned to have an elevated disease recurrence risk.

## RESULTS

### MALAT1 levels are altered in breast cancer subtypes

**Figure 1:**
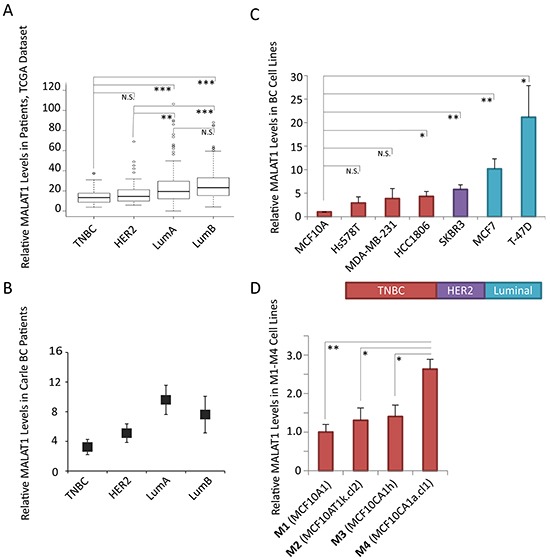
Relative levels of MALAT1 in breast cancer tissue samples and cell lines of different subtypes **A.** Box-plot data depicting the relative levels of MALAT1 in breast cancer patient samples from different subtypes, obtained from the TCGA microarray data set. TNBC (n=77), HER2+ (n=54), Luminal A (n=232), Luminal B (n=129) **B.** RT-qPCR analyses to determine the relative levels of MALAT1 in Carle Cancer Center patients; TNBC (n=5), HER2+ (n=5), Luminal A (n=3), Luminal B (n=3). Bars indicate the average of three biological repeats ±SEM. **C.** RT-qPCR analyses to determine the relative levels of MALAT1 in cell lines of different breast cancer subtypes, bars indicate the average of three biological repeats ±SEM **D.** RT-qPCR analyses to determine the relative levels of MALAT1 in M1-M4 cell lines, two-tailed Student t-test was performed, * and ** indicate p-value<0.05 and <0.01, respectively.

To investigate the contribution of MALAT1 in cancer progression in different breast cancer (BC) subtypes, we determined MALAT1 levels in various BC subtype samples using the Cancer Genome Atlas (TCGA) microarray data set, containing 492 BC patient samples of various subtypes [2]. Among different subtypes, MALAT1 showed the highest levels in luminal patients while the lowest levels are observed in ER negative patient samples of the HER2 or basal-like/TNBC molecular subtypes (Figure 1A). Further, by performing RT-qPCR, utilizing RNA from a small independent cohort of patient samples from Carle Cancer Center, we confirmed the TCGA microarray data, demonstrating elevated levels of MALAT1 in patient samples of luminal subtype compared to HER2+ and TNBC subtypes (Figure 1B).

A better understanding of MALAT1 function in different subtypes could be obtained by manipulating the levels of MALAT1 in breast cancer cell lines of various subtypes. We examined the levels of MALAT1 in a human non-tumorigenic breast epithelial cell line (MCF10A) and BC cell lines from different subtypes (TNBC subtype: MDA-MB-231, Hs578T, HCC1806; HER2^+^ subtype: SKBR3; luminal subtype: MCF7, T-47D) (Figure 1C). RT-qPCR results revealed elevated levels of MALAT1 in T-47D cells, which is of luminal B subtype cells. In general, compared to other subtypes of BC cells, TNBC cells contain lower levels of MALAT1. In order to study the function of MALAT1 in an isogenic cell line background, we expanded our screening to a well characterized and widely reported isogenic breast cancer progression cell line series, including four cell lines named M1, M2, M3, and M4 [59–63]. These four cell lines are of basal-like/TNBC subtype (basal-like) and are all derived from non-tumorigenic MCF10A mammary epithelial cells: benign MCF10A (M1), transformed H-Ras-transfected MCF10A; MCF10AT (M2), tumorigenic but low metastatic MCF10CA1h (M3), and highly tumorigenic/metastatic MCF10CA1a.cl1 (M4) [59–63]. In particular, M2 cells were isolated from tumors in nude mice that were injected with H-Ras overexpressing M1 cells, whereas both M3 and M4 cells were isolated from tumors, which were developed in mouse xenografts after the nude mice were injected with M2 cells. Thus, M1-M4 system includes a cancer progression spectrum from a relatively normal breast epithelial cell line (M1) to a highly tumorigenic and metastatic breast cancer cell line (MCF10CA1a.cl1 or M4). In this model, we observed elevated levels of MALAT1 in the highly metastatic M4 cell line compared to the other three cell lines (M1, M2 and M3), supporting the potential involvement of MALAT1 in driving metastasis in basal-like TNBC cells (Figure 1D).

### Role of MALAT1 in tumor progression is not limited to luminal subtype

**Figure 2:**
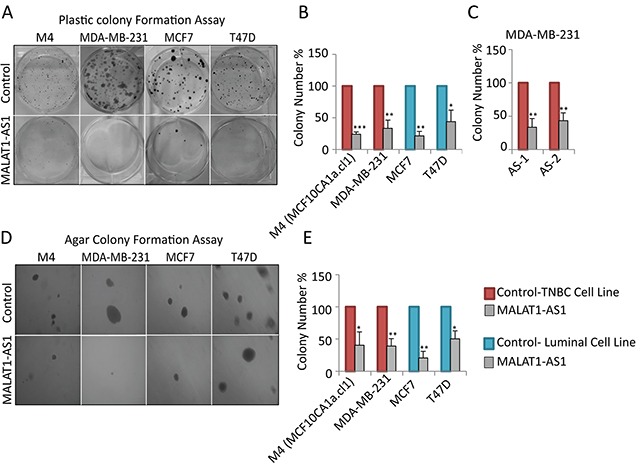
Depletion of MALAT1 in breast cancer cells decreases cell proliferation and anchorage-independent colony formation **A-B.** Depletion of MALAT1 using DNA antisense oligonucleotides decreases cell proliferation in different breast cancer subtypes. Cell proliferation is evaluated by clonogenic (plastic colony formation) assay **C.** Plastic colony formation assay in MDA-MB-231 cells that are treated with control or two different MALAT1-specific antisense oligonucleotides. **D-E.** MALAT1 depletion decreases anchorage-independent growth in different cell lines. Data represented in A-E are from three biological repeats. Error bar indicates SEM.

Next, we investigated the role of MALAT1 in tumor progression by depleting MALAT1 in BC cell lines of various subtypes and assayed the effect on various cancer cell attributes; proliferation, anchorage-dependent growth by clonogenic (plastic colony formation) and anchorage-independent soft agar colony assays. Since MALAT1 shows its highest expression in luminal patients/cells, we first depleted MALAT1 using modified antisense oligonucleotides (ASOs) in luminal cell lines; T47D and MCF7 cells (Figure 2A-2B, Suplementary Figure S1A), and tested the effect on long-term anchorage-dependent colony formation in plastic plates. Luminal cells upon depletion of MALAT1 showed reduction in cell proliferation (Figure 2A-2B). Our observations are in agreement with the recent data, showing the involvement of MALAT1 in tumor progression in luminal cells [64]. Next, we performed similar experiments in TNBC cell lines, including MDA-MB-231 (MB-231) and M4 (MCF10CA1a.cl1) cells. MALAT1 depletion in TNBC cells using two independent ASOs (AS-1 & AS-2) against MALAT1 resulted in dramatic decrease in cell proliferation (Figure 2A-2C, Supplementary Figure S1A). Similar results were also obtained in MDA-MB-231 cells when cells were depleted of MALAT1 using independent siRNAs instead of DNA-based ASOs (Supplementary Figure S2). Next, we tested the involvement of MALAT1 in the tumorigenicity in BC cells by performing anchorage-independent soft agar colony formation assay. We consistently observed decreased ability for MALAT1-depleted cells to grow in soft agar-coated plates, both for luminal and TNBC subtypes, indicating that MALAT1 plays crucial roles in the tumorigenic properties of the BC cells (Figures 2D, 2E). These results indicate that MALAT1 plays vital roles in the tumor progression in various subtypes of BC.

### MALAT1 regulates metastasis in TNBC cells

**Figure 3:**
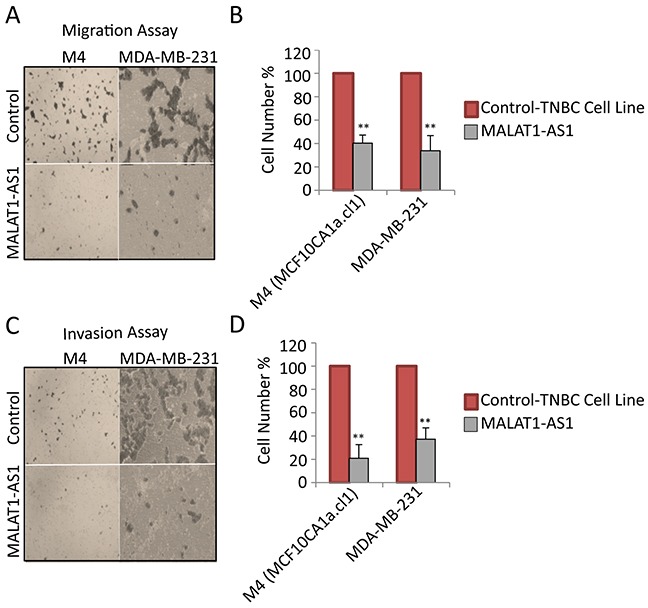
MALAT1 depletion decreases migration and invasion in metastatic TNBC cells **A.** Representative micrographs showing the migration of cells upon control and MALAT1-depletion. **B.** Migration assay quantification from three biological repeats and error bar indicates SEM. **C.** Representative micrographs showing invasion of cells upon control and MALAT1-depletion. **D.** Invasion assay quantification from three biological repeats. Error bar indicates SEM.

Luminal cells such as MCF7 and T-47D generally display weak metastatic properties under in vitro and in vivo conditions. Therefore, to investigate the involvement of MALAT1 in metastasis, we performed in vitro metastatic assays in control and MALAT1-depleted TNBC cell lines (MDA-MB-231 and M4), both of which are previously shown to be highly metastatic in nature [65]. We evaluated both migratory and invasive properties of control and MALAT1-depleted cells using migration and invasion transwell chamber assays in vitro. MALAT1 depletion resulted in decrease in the migratory (Figure 3A-3B) and invasive (Figure 3C-3D) properties of both these cell lines. Similar results were observed using another independent set of ASO as well as siRNAs, against MALAT1 (Supplementary Figure S3, S1B).

Next, we examined whether the overexpression of MALAT1 could induce proliferation, tumorigenicity and metastasis in BC cell lines. To test this, we stably expressed wild type mouse Malat1 cDNA in non-tumorigenic MCF10A cells using retro- or lenti-viral mediated transduction (Supplementary Figure S4A). MCF10A cells form three-dimensional acini when grown in 3D culture in Matrigel-a basement membrane-like extracellular matrix [66]. Such organotypic three dimensional MCF10A cell cultures mimic several vital aspects of mammary gland morphogenesis and architecture, and is routinely used to study mammary gland biology in vitro [66–69]. We grew control and MALAT1-overexpressing MCF10A cells under 3D-conditions and analyzed their growth. MALAT1-overexpressing MCF10A cells showed increased proliferation compared to control cells, as observed by increased size in acini structure (Supplementary Figure S4B) as well as by enhanced labeling by Ki67 antibody (Supplementary Figure S4C).

**Figure 4:**
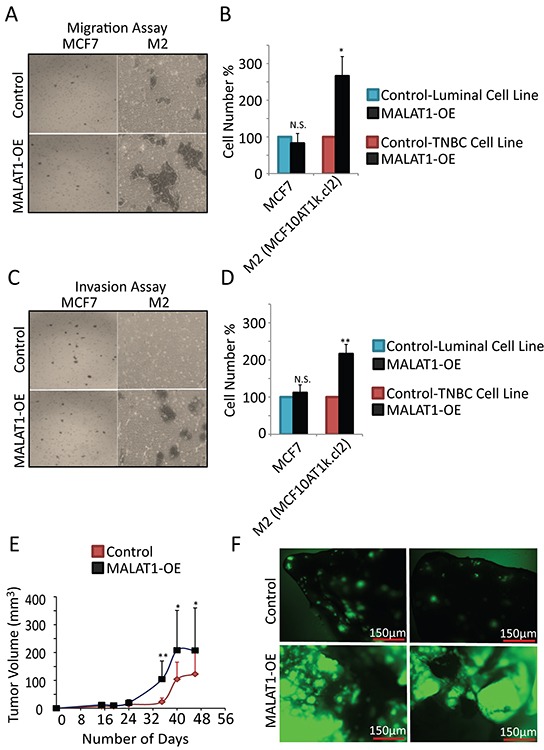
MALAT1 overexpression accelerates metastasis in TNBC cells **A.** Representative micrographs showing the migration of MCF7 and M2 cells upon control and MALAT1-overexpression. **B.** Migration assay quantification from three biological repeats. Error bar indicates SEM. **C.** Representative micrographs showing invasion of MCF7 and M2 cells upon control and MALAT1-overexpression. **D.** Invasion assay quantification from three biological repeats. Error bar indicates SEM. **E.** Pools of GFP-labeled MDA-MB-231 and MDA-MB-435s cells were transduced with the indicated lenti virus encoding MALAT1 or an empty vector. After puromycin selection, cells (2×10^6^) were injected subcutaneously into NOD-SCID mice (n=10). Tumor volume was measured bi-weekly. **F.** 6 weeks after injection, mice were sacrificed and lungs from mice injected with the GFP-labeled MDA-MB-435s cells were visualized to detect GFP-labeled metastasis.

Next, we analyzed the involvement of MALAT1 in the metastatic properties of cells. In our hands, MALAT1 overexpression in luminal cell line, MCF7 showed no effect on migration and invasion (Figure 4A-4D, Supplementary Figure S1C). At the same instance, MALAT1-overexpressed weakly tumorigenic M2 cells showed increased migration (Figure 4A-4B) and invasion (Figure 4C-4D). This result suggests that under in vitro conditions, MALAT1 level may be more critical in regulating metastasis in TNBC cells compared to luminal subtype.

We also determined proliferation and tumorigenic properties of MDA-MB-435s cell line upon MALAT1 overexpression. The exact origin of MDA-MB-435s cells is under constant debate [70]. MDA-MB-435s was initially thought to be of breast cancer origin [71], and is still extensively used in breast cancer research [72–80]. For example, global gene expression analyses indicate that MDA-MB-435s cells show similar gene signature to that TNBC/basal-like BC subtype [81]. However, other independent studies indicate that MDA-MB-435s cells could be originally derived from melanoma cells [82–84]. In general, MALAT1-overexpressed MDA-MB-435s cells showed increased proliferation under normal as well as under serum-starved conditions (Supplementary Figure S5A-S5B). In addition, MALAT1-overexpressed cells also showed increased number of colonies in anchorage-independent soft agar assay (Supplementary Figure S5C). Furthermore, MALAT1-overexpressed cells showed decreased sensitivity to anisomycin-induced apoptosis (Supplementary Figure S5D), condition that is similar to what is observed in the case of oncogene-activated cells [85]. Experiments utilizing serum starvation and anisomycin –induced apopotosis provide insights into the oncogenic potential of MALAT1 in the context of apoptosis of the breast cancer cells. For example, malignant cancer cells are known to be more resistant to apoptosis, thus reduced apoptosis after exposure to anisomycin in reduced serum condition points towards the oncogenic potential and anti-apoptotic properties of MALAT1. All these results indicate that irrespective of the cell of origin, MALAT1 enhances the tumorigenic properties of MDA-MB435s cells.

To investigate the oncogenic and metastatic potential of MALAT1 in vivo, we injected MDA-MB-435s cells with GFP reporter, over-expressing MALAT1 or the empty vector subcutaneously into NOD-SCID mice. We found that MALAT1 overexpression further induces the oncogenic property of MDA-MB-435s cells as they formed large tumors in mice (Figure 4E). This result suggests that MALAT1 was capable of imparting greater oncogenic potential in MDA-MB-435s cells. To examine the metastatic potential of MALAT1, we looked at the metastatic effect of MALAT1 overexpression in lungs of mice injected with GFP +ve MDA-MB-435s cells overexpressing full length human MALAT1 or the empty vector. We found that over-expression of MALAT1 significantly increased the metastasis capacity of MDA-MB-435s cells (Figure 4F). Altogether, these results confirm that under in vivo conditions, over expression of MALAT1 enhances the tumorigenic and metastatic properties of cancer cells.

We have previously demonstrated the involvement of MALAT1 in regulating the localization and activity of several pre-mRNA splicing factors, including the SR family of splicing factors such as SRSF1 [51, 86]. For example, MALAT1-depleted human cells showed changes in the alternative splicing of pre-mRNAs of several genes that are involved in tumor progression and metastasis [52, 53, 64, 86]. We therefore determined whether overexpression of MALAT1 influences the alternative splicing of key cancer-associated genes in MD-MBA-435s cells. Particularly, we looked at the changes in the SRSF1-mediated alternative splicing in these cells. RT-PCR-based splicing assays revealed that MALAT1-overexpressed cells showed changes in the SRSF-mediated alternative splicing of pre-mRNAs of several cancer–associated genes, including *TEAD, BIN* and *BIM* (Supplementary Figure S5E). These results indicate that even in breast cancer cells differential levels of MALAT1 could alter alternative splicing of key oncogenic gene mRNAs, preferentially through modulating the activity of SR-splicing factors, such as SRSF1.

### MALAT1 regulates the expression of genes involved in cell cycle progression and epithelial-to-mesenchymal transition in BC cells

**Figure 5:**
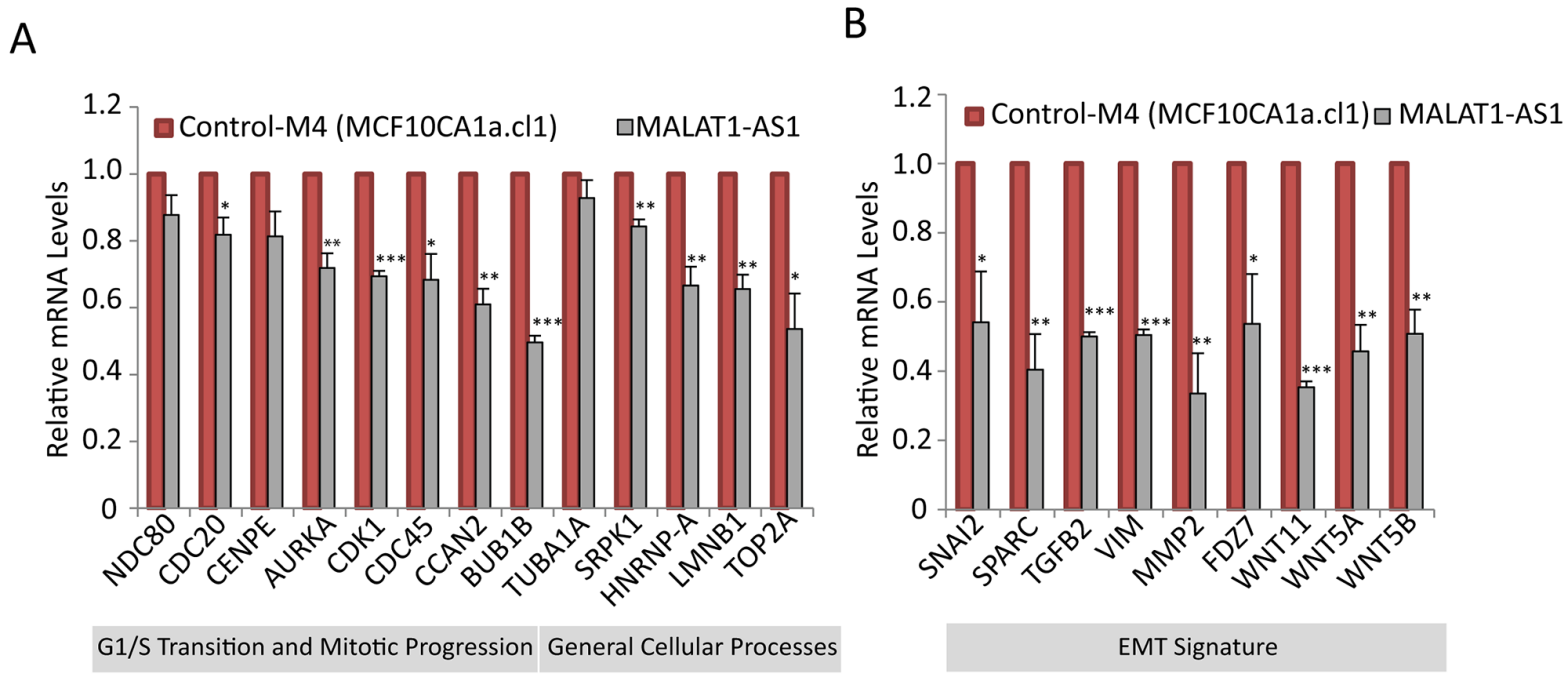
MALAT1 regulates the expression of genes involved in cell cycle progression and EMT signature RT-qPCR analyses to detect relative levels of mRNA of genes that are involved in cell cycle **A.** and EMT **B.** in control and MALAT-depleted M4 cells.

Next, we attempted to identify the downstream target genes of MALAT1, the altered expression of which in MALAT1-expression altered cells, contributes to changes in cell proliferation, tumor progression and metastasis. We had previously reported that the levels of MALAT1 are regulated during the cell cycle, and MALAT1 modulates the expression of a large number of cell cycle-regulated genes in human lung fibroblasts [52]. To determine if MALAT1 regulates the expression of similar set of cell cycle genes in breast cancer cells as well, we performed RT-qPCR to quantify the mRNA levels of several of these genes in control and MALAT1-depleted M4 cells (Figure 5A). MALAT1-depleted M4 cells showed down regulation of several of the candidate cell cycle genes, several of which are known to play vital roles in G1/S and mitotic progression. Next, we determined whether MALAT1 overexpression in non-tumorigenic M2 cells would induce the expression of these cell cycle genes. We consistently observed upregulation of a few (*CENPE and TUBA1A*) but not all of the cell cycle-regulated genes in MALAT1 overexpressed M2 cells (Supplementary Figure S6A). Several of the other candidate mRNAs showed only moderate increase in their levels upon MALAT1 overexpression.

To determine changes in the expression of genes involved in metastasis in MALAT1-depleted BC cells, we used an EMT (Epithelial-mesenchymal transition) profiler qPCR array (Qiagen). EMT is a process in which epithelial cells lose epithelial cell-cell adhesions, gain mesenchymal properties and become increasingly migratory and invasive in nature [87]. Under physiological conditions, EMT happens during mesoderm and neural tube formation during embryonic development [88]. EMT is also observed during organ fibrosis and wound healing where cells lose adhesion properties and migrate to repair the wound [89]. Under pathological circumstances, EMT is involved in the initiation of cancer metastasis where tumor cells depart the primary tumor site and invade and migrate to other tissues, thereby expanding their territories [90]. EMT profiler assay in control and MALAT1-depleted M4 cells revealed that the expression of a significant number of EMT genes was affected upon MALAT1 depletion [Supplementary Table S1]. Some of the key down regulated genes in MALAT1-depleted samples include *SPARC, SNAI2, MMP2, TGFB2, WNT5B, VIM, FDZ7, WNT11 and WNT5A*. These genes are known to be up regulated during EMT. We further quantified the mRNA levels of several of these genes in control and MALAT1-depleted M4 cells by RT-qPCR, and observed consistent results (Figure 5B). Furthermore, we examined the level of a panel of these mRNAs upon MALAT1 depletion in both M4 and MDA-MB-231 cells using two independent ASOs (AS-1 and AS-2) and observed similar results (Supplementary Figure S7A-S7B). We also determined the mRNA levels of the EMT genes in MALAT1-overexpressed M2 cell line. We consistently observed increased expression of genes such as *VIM and WNT5A* in MALAT1-overexpressed cells (Supplementary Figure S6B). The expression of genes such as *IL1RN, CDH1, RON, FGFBP1, DSP, OCLN, CAV2, NUDT13, PPPDE2 and RGS2* are known to be down regulated during EMT. Consistently, MALAT1-depleted M4 cells showed increased mRNA levels of these genes (Supplementary Figure S8). Deregulation of several EMT genes upon altered expression of MALAT1 in metastatic BC cells suggests that MALAT1 could regulate metastasis through regulating the expression of key EMT genes.

### Elevated MALAT1 levels correlate with poor prognosis in LN- patients of TNBC and HER2+ subtypes

**Figure 6:**
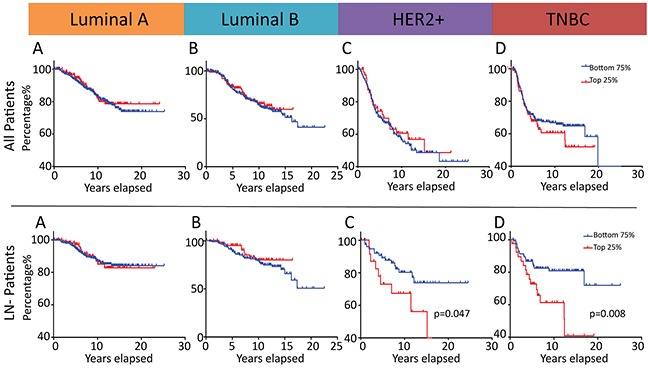
MALAT1 is a potential prognostic marker in patients diagnosed with ER negative lymph node negative breast cancer Prognostic significance of MALAT1 expression in breast cancer with regard to disease-specific survival (DSS) in an independent 1992 patient Microarray Dataset [91]. Kaplan-Meier analysis of DSS in patients diagnosed with tumors of Luminal A, Luminal B, HER2 or basal-like/TNBC subtypes with a subtype-specific top quartile cutoff **A-D.** Kaplan-Meier analysis of DSS in lymph node–negative (LN-) patients diagnosed with tumors of Luminal A, Luminal B, HER2 or basal-like/TNBC subtypes with a subtype-specific top quartile cutoff **E-G.** All p-values are two-sided.

We next sought to examine whether the above delineated role of MALAT1 in regulating aggressive cellular traits and mediating tumor progression and metastasis has a measurable prognostic impact in human breast cancer patients. When patients diagnosed with all BC molecular subtypes (Luminal A/B, HER2 and basal-like/TNBC) were analyzed together, there were no statistically significant differences in Disease-Specific Survival (DSS) between patients whose tumors displayed high or low MALAT1 expression, irrespective of the specific percentile cutoff value employed (data not shown). When DSS was analyzed in this cohort within each subtype (Luminal A/B, HER2 and basal-like/TNBC), MALAT1 expression level still was not associated with any statistically significant difference with respect to DSS, irrespective of the specific percentile cutoff value employed (Figure 6A-6D). Only when we examined the LN negative subset of patients within each molecular subtype did significant differences in DSS become apparent between low and high MALAT1 expression groups. This is of great clinical significance as disease recurrence and metastasis in patients diagnosed with cancers of ductal origin (e.g. adenocarcinomas), in the absence of lymph node involvement, is strongly suggestive of the less common hematogenous route of dissemination that is more commonly encountered in the case of tumors of mesenchymal origin (e.g. sarcomas). In univariate analysis, DSS was significantly worse in tumors from patients diagnosed with ER negative lymph node negative breast cancer which displayed the top quartile of subtype-specific MALAT1 expression (HR= 2.32, 95%CI 1.02-5.31, P = 0.047 in HER2+ LN-, HR=2.46, 95%CI 1.27-4.78, P = 0.008 in basal-like LN-, respectively) (Figure 6G-6H). This pattern was not observed in the case of Luminal A LN- or Luminal B LN- patients thereby suggesting a molecular subtype context specific effect of MALAT1 on mediating tumor progression and metastasis (Figure 6E-6F). Surprisingly, such a correlation was not observed in the case of LN+ patients (data not shown). Furthermore, multivariable analysis confirmed MALAT1 to have independent prognostic significance in the basal-like lymph node negative patient subset (HR=2.64, 95%CI 1.35-5.16, p=0.005). While similar multivariable analysis in the HER2+ LN- group (HR=2.28, 95%CI 1.00-5.22, p=0.052) did not emerge to statistical significance to confirm the prognostic importance of MALAT1 expression in this subgroup of patients (that had been noted on the initial univariate analysis), this effect is likely secondary to limitations of the sample size and merits investigation in a larger breast cancer patient cohort.

### MALAT1 shows subtype-specific correlation with EMT genes in breast cancer patients

**Figure 7:**
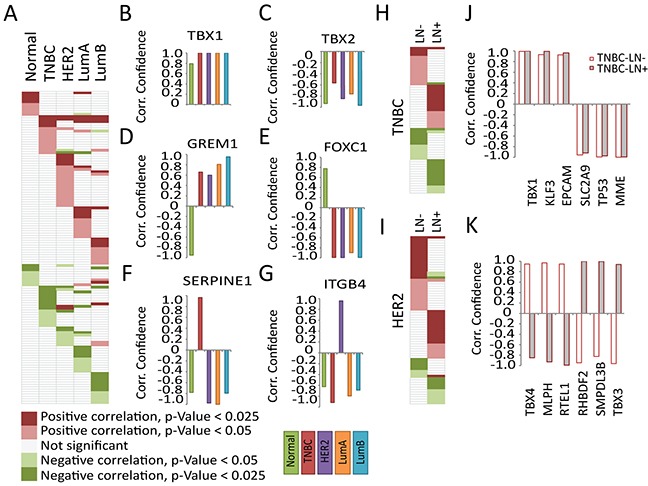
Subtype-specific expression correlation of MALAT1 and EMT signature genes in breast cancer patients **A.** The EMT signature genes showing potential correlation with p-value<0.05 in different subtypes, the gene names not shown due to space limit (for detailed information see Supplementary Table S2). Representative genes showing potential correlation in all subtypes **B.** *TBX1*/ILMN_2248112 (gene name/probe number) and **C.** *TBX2*/ILMN_1792256. Representative genes with different pattern in normal and patient samples **D.** *GREM1*/ILMN_2124585 and **E.** *FOXC1*/ILMN_1738401. Representative genes showing subtype-specific correlation **F.** *SERPINE1*/ILMN_1744381 and **G.** *ITGB4*/ILMN_2317543. The EMT signature genes showing potential correlation with p-value<0.05 in LN- and LN+ pool in **H.** TNBC and **I.** HER2 patients (for detailed information see Supplementary Table S2). Representative genes showing correlation in LN- and LN+ TNBC samples **J.** with similar pattern; *TBX1*/ILMN_2248112, *KLF3*/ILMN_1670245, *EPCAM*/ILMN_2160209, *SLC2A9*/ILMN_1668312, *TP53*/ILMN_1779356, *MME*/ILMN_1786319 and **K.** with different pattern; *TBX4*/ILMN_1745827, *MLPH*/ILMN_1795342, *RTEL1*/ILMN_1709538, *RHBDF2*/ILMN_1735792, *SMPDL3B*/ILMN_1719660, *TBX3*/ILMN_1713449. Same color code in J-K.

Based on the data obtained from BC cell lines and BC patients, MALAT1 facilitates tumor progression differently in various BC subtypes. We therefore hypothesize that the subtype-specific function of MALAT1 could potentially be due to the role of MALAT1 in regulating the expression of specific subset of genes in each of the subtype. To find potential target/s, we explored the expression correlation between MALAT1 and co-expressed genes in patients using data from the Curtis et al. clinical dataset [91]. To identify potential partners/targets, we first curated the literature to derive a list of several EMT-associated genes and other transcription factor genes (250 genes ~ 459 hits) with potential roles in tumor development and metastasis. We then calculated Pearson Correlation Coefficient (PCC) as a measure for correlation between the expression of MALAT1 and the gene of interest [Supplementary Table S2]. Then, we calculated the p-value and confidence level for each correlation. We identified genes whose mRNA levels are positively and negatively correlated with MALAT1 (Figure 7A; p-value<0.05). The detailed gene list is documented in Supplementary Table S2. Our analyses identified several patterns of correlation between MALAT1 and specific set of genes. The expression of few genes was correlated with MALAT1 in patient samples from all of the subtypes (Figure 7B-7C]. For example, *TBX1* levels were correlated significantly with MALAT1 levels in all of the tested samples (Figure 7B). *TBX1* gene encodes for a T-box transcription factor 1, and its elevated level is associated with breast tumor development [92]. TBX2 is another member of this family, which showed negative correlation with MALAT1. Genes such as GREM1 and FOXC2 displayed opposite correlation trend in normal and patient samples regardless of the subtype tested (Figure 7D-7E). Interestingly, for another subset of genes, the correlation with MALAT1 was subtype specific. SERPINE1 and ITGB4 are two examples of genes, which showed subtype-specific correlation in their levels with MALAT1 (Figure 7F-7G). In addition, MALAT1 level was also correlated with specific set of genes in each LN- and LN+ pools (Supplementary Table S2) (Figure 7H-7I; data shown for TNBC and HER2). For example in the case of TNBC subtype, genes such as *TBX1, KLF3, EPCAM, SLC2A9, TP53* and *MME* showed similar pattern in both LN- and LN+ pools (Figure 7J). On the other hand, genes such as *TBX4, MLPH, RTEL1, RHBDF2, SMPDL3B* and *TBX3* correlated with MALAT1 differentially in LN- and LN+ patients (Figure 7K). These correlation analyses in patient samples imply that MALAT1 could potentially regulate the expression of key genes involved in tumor progression and/or metastasis in BC cells of specific subtype or of different LN status.

## DISCUSSION

MALAT1 is a highly abundant and conserved lncRNA, and is localized to the nuclear speckle sub-compartment. Earlier studies indicate that MALAT1 modulates transcription and pre-mRNA processing of a large set of genes [51–54, 64]. MALAT1 is deregulated in several tumors, and is potentially of significant clinical importance. In this study, we have determined the involvement of MALAT1 in different subtypes of breast cancer. Based on the gene expression data in BC tissues and in cell lines, MALAT1 levels were consistently high in luminal subtype and relatively low in TNBC subtypes. This implies that MALAT1 might play a crucial role in tumor progression in luminal subtype over TNBC subtype. However, our functional analysis revealed that though MALAT1 is less abundantly expressed in TNBC subtype compared to luminal subtypes, it plays crucial role in regulating the expression of key genes that are involved in tumor progression and metastasis in TNBC cells.

The molecular signature of gene expression in TNBC is one of the most unique among all of the intrinsic subtypes of breast cancers [2, 7, 93, 94]. In addition, the age of disease incidence, and the risk factors associated with TNBC patients are different and sometimes opposite when compared to other subtypes of BC [95]. It was hypothesized that the various subtypes of breast cancer could be originated from different cell types, and some of these differences could be attributed to different cell origins [96]. It is possible that differential levels of MALAT1 in various subtypes of BC could also be attributed to various cell types from where the tumor is derived.

To identify the role of MALAT1 in breast tumor progression, we determined the potential changes in the levels of the RNA of interest in a series of BC cell lines of isogenic background. The M1-M4 isogenic cell line model provides an ideal tool to answer such questions. All of the four cell lines are derived from MCF10A, which is a well-established non-tumorigenic mammary epithelial cell line [97]. The properties of M1 to M4 cells reflect a natural progression of cancer from a non-tumorigenic (M1), to hyper-proliferative, non-tumorigenic (M2), to tumorigenic, non-metastatic (M3), and finally to highly tumorigenic and metastatic (M4) state. Further, PAM50 analyses indicate that M1-M4 cells show basal-like/TNBC gene signature (data not shown), making them an ideal model to study the involvement of a gene in tumor progression and metastasis in TNBC subtype. We observed elevated levels of MALAT1 in M4 cells compared to M1-M3. Furthermore, MALAT1 depletion in M4 and overexpression in M2 cells decreased and increased tumorigenic and metastatic properties of these cells, respectively. This supports our hypothesis that MALAT1 plays an active role in regulating tumor progression and metastasis in TNBC cells.

To determine whether MALAT1 expression in breast cancer is capable of exerting prognostically significant effects, we compared DSS (disease-specific survival) between patients grouped according to high or low MALT1 expression. Elevated MALAT1 levels were found to be associated with decreased DSS in ER negative, lymph node negative patients of the HER2 and basal-like or TNBC molecular subtypes. Multivariate analysis further confirmed the independent prognostic significance of MALAT1 expression in the basal-like lymph node negative patient subset. This further confirms our hypothesis that the functional effect of MALAT1 on tumor progression and metastasis is context dependent upon the molecular subtype and clinical phenotype of the cancer, and not merely based upon its expression level alone. We propose that the functional significance of MALAT1 as a metastasis driver and its potential use as a prognostic marker is most promising for those patients diagnosed with ER negative, lymph node negative breast cancer. Our findings are of particular clinical significance as MALAT1 levels in breast cancer samples could be used to predict future onset of metastatic disease in LN- patients who might otherwise be perceived to be at a low risk for metastasis. This information could influence treatment recommendations in favor of administering adjuvant chemotherapy (with the therapeutic rationale of reducing or curbing the risk of such future metastatic events) in this group of patients who might otherwise not be offered such therapy. Our results thus support the potential use of monitoring MALAT1 expression level as a predictor of tumor recurrence and metastasis in patients diagnosed with ER negative lymph node negative BC. Subsequent clinical validation studies to confirm the prognostic predictive ability of MALAT1 expression, if successful may find clinical utility in guiding treatment recommendations to undergo systemic adjuvant chemotherapy in a group of BC patients who might otherwise mistakenly be perceived to be at low risk of disease recurrence.

We observed that TNBC cells that were either overexpressing or depleted of MALAT1 showed altered expression of genes that are involved in cell cycle, tumor progression and EMT. In addition, MALAT1 and mRNA correlation analysis in Curtis patient data sets identified several EMT genes the mRNA levels of which were potentially correlated with MALAT1 levels in specific breast cancer intrinsic subtypes. Particularly, we observed that each BC subtype contains unique set of genes, the mRNA levels of which were specifically correlated with MALAT1 levels only in one subtype but not in others. Such data support the view that MALAT1 might contribute to regulating different network of genes not only in different cancers but also in different subtypes or LN − or LN + types of a specific cancer such as breast cancer. A very recent study reported that in luminal B breast cancer model system, depletion of MALAT1 did not alter the expression of genes involved in EMT, even though the cells showed defects in tumor progression and metastasis [64]. On similar lines, several earlier studies have reported that in various cancers MALAT1 influences tumor progression and metastasis by controlling distinct cellular pathways [57, 58]. For example, MALAT1 is shown to modulate the expression of several of the cell motility and metastasis-associated genes at the transcriptional and/or post transcriptional level in lung cancer [53, 98]. On the other hand, MALAT1 in bladder cancer cells is reported to associate with a polycomb repressive component, SUZ12. Consequently, this association influences the differential expression of *N-Cadherin* and *E-cadherin*, thereby facilitating EMT [99]. In cervical cancers, depletion of MALAT1 induces the expression of pro-apoptotic genes and inhibits the activity anti-apoptotic genes [100]. Finally, in colon cancer cells, MALAT1-depletion is reported to decrease the nuclear localization of β-catenin thereby inhibiting wnt/β-catenin signaling [101]. In luminal breast cancer cells, a whole-genome analysis identified several mutations in SRSF1-binding region of MALAT1. This may affect the SRSF1-mediated pre-mRNA splicing in these cells since MALAT1 is known to interact with SRSF1 and regulates the activity of SR proteins [39, 51, 52, 86]. A very recent study from the Spector laboratory also pointed out the involvement of MALAT1 in regulating the expression and pre-mRNA splicing of genes that are involved in differentiation and pro-tumorigenic pathways in breast cancer model system [64]. All this data further supports our argument, that MALAT1-mediated changes in gene expression are cell or tissue type specific. In addition, our correlation analysis has provided vital hints about the potential targets and interacting partners of MALAT1 in specific subtypes of BC.

The expression of MALAT1 in a cell is controlled both at transcriptional and post-transcriptional level [58]. For example, histone H3K9 (histone 3 lysine 9) demethylase, JMJD1A demethylates H3K9 at the MALAT1 promoter, resulting in the induction of MALAT1 expression [102]. We have recently demonstrated that a natural antisense lncRNA from *MALAT1* locus that we named as TALAM1 positively regulates the stability of MALAT1 RNA [103]. In addition, several miRNAs negatively regulate MALAT1 by facilitating its degradation [58]. For example, a very recent study has reported mutual negative correlation between MALAT1 and miR-1 in TNBC model [41]. This study revealed that in BC cells MALAT1 facilitated the expression of *Slug*, a pro-EMT gene, by negatively regulating the interaction of miR-1 and slug mRNA. The complex regulatory layers governing cellular levels of MALAT1 further implicate its dose-dependent and context-specific function. MALAT1 is a tightly regulated and multifunctional lncRNA, which controls key gene networks by fine-tuning both transcription and alternative splicing of specific cancer-associated genes in different subtypes of breast cancer cells. Future studies will confirm such interactions, and will also decipher the mechanistic significance of such interactions between MALAT1 and specific set of genes in causing tumor progression and/or metastasis.

## MATERIALS AND METHODS

### Cell culture

MDA-MB-231 and MCF7 cells were cultured in DMEM supplemented with 1mM sodium pyruvate, 100U/mL penicillin, 100 μg/mL streptomycin and 10% fetal bovine serum. M4 cells were cultured in DMEM/F12 medium supplemented with 100 U/mL penicillin, 100 μg/mL streptomycin and 5% horse serum. M1-M3 cells were cultured in DMEM/F12 medium supplemented with 100 U/mL penicillin, 100 μg/mL streptomycin, 20ng/mL EGF (epidermal growth factor), 0.5 μg/mL Hydrocortisone, 100ng/mL Cholera toxin, 10 μg/mL insulin and 5% horse serum. SKBR3 cells were cultured in McCoy's 5a Medium supplemented with 100U/mL penicillin, 100 μg/mL streptomycin and 10% fetal bovine serum. T-47D cells were cultured in RPMI-1640 Medium supplemented with 0.2 Units/ml insulin, 100U/mL penicillin, 100 μg/mL streptomycin and 10% fetal bovine serum.

### RNA extraction and RT-qPCR analysis

Total cellular RNA was extracted from cells using Trizol reagent (Invitrogen) following manufacturer's protocol. RNA was reverse transcribed into cDNA using Random hexamers and Multiscribe reverse transcriptase (Applied Biosystems). Quantitative RT-PCR was performed using StepOne Plus system. (Applied Biosystems) and primers listed in Supplementary Table S3.

### MALAT1 knockdown/overexpression experiments

MALAT1 depletion was carried out by transfecting cells either using antisense oligonucleotide (ASO) or siRNAs against MALAT1 [50]. Briefly, cells were transfected with control or MALAT1-specific ASOs (100 nM final con.) or siRNAs (40-50 nM con) for two rounds with a gap of 24 hrs using Lipofectamine RNAiMax reagent (Invitrogen, USA). For overexpression, full-length mouse or human Malat1/MALAT1 were expressed using retro- or lenti-virus-mediated transduction, and stable lines were selected using puromycin selection. Empty vector transduced cells were used as control.

### Alternative pre-mRNA splicing assay

Pools of MDA-MB-435s cells were transduced with the indicated lentiviruses encoding MALAT1 or an empty vector. After puromycin selection, cells were lysed and RNA was isolated. Splicing patterns of SRSF1 target genes (BIM, BIN-1 and TEAD-1) were examined using the indicated isoform specific primers. GAPDH mRNA was used as control. Primers are listed in Supplementary Table S3.

Total RNA was extracted with Tri-reagent (Sigma) and 1 μg of total RNA was reverse transcribed using M-MLV reverse transcriptase (Promega). PCR was performed on1/10 volume 2μl of the cDNA, in 25 μl reactions containing 12.5 μl of PCR Mix (Kapa Biosystem), 1.25 μl of 10 μM forward and Reverse Primer, 1.8 μl of DMSO. PCR conditions were as follows 95°C for 3 minutes, then 34 cycles of 95°C for 15 sec, 60°C for 15 sec, 72°C for 45 sec followed by 10 minutes at 72°C. PCR products were separated on 2% agarose gel.

### Proliferation assay

Cells were seeded in 96 well plates. After stipulated time, they were fixed with 2.5% Glutaraldehyde and stained with 1% methylene blue in 0.1M Borate Buffer. The absorbance of the acid extracted stain with 0.1N HCl was measured on a plate reader (Bio-rad) at 655nM.

### Anisomycin-mediated cell death assay

MDA-MB-435s cells were transduced with lentiviruses encoding an empty vector or full length human MALAT1. Following Selection, 0.2 × 106 cells were seeded per well in six well plates. 24 hours later, cells were incubated with 1 μM Anisomycin in 0.1% serum DMEM medium for 24 hours. Medium and PBS washes were collected together with cells trypsinized from each well into 15ml tubes and centrifuged at 1500RPM for 5 min. Cells were washed with PBS and after another centrifugation were resuspended in 50 μl of HEPES Buffer. 10 μl of the cell suspension was mixed with 10 μl of 4% trypan blue solution and live/dead cells were counted using a Bio-Rad TC-10 Automated Cell Counter.

### Plastic colony formation assay (anchorage-dependent growth)

1000 cells (control, MALAT1-depleted or – overexpressed) per well were seeded in a 6-well plate. After 7-14 days, cells were washed, fixed with ice-cold methanol and stained with crystal violet 0.05% in MeOH:H2O with ratio of 1:9. Colony numbers were counted per each well.

### Agar formation assay (anchorage-independent growth)

In a 12-well plate, 2ml medium with 0.5% low-melt agarose was laid down and the plate was placed at 4°C for 20 minutes. Next, 2500 cells (control, MALAT1-depleted or – overexpressed) were resuspended in 1.5 ml medium with 0.35% low-melt agarose was laid down on the bottom layer. The plate was placed at 4°C for another 20 min. Then, it was transferred to incubator at 37°C, 5% CO2 and cultured for 18-30 Days. Cells were stained with crystal violet 0.005% in MeOH: H2O: Acetic Acid with ratios of 5:4:1 respectively.

### Migration assay

Migration assay was carried out using 8μM transwell migration chambers (Corning, Cat# 354578). Cells were starved in serum-free medium for 5-6 hrs, then trypsinized and resuspended in serum-free medium and seeded in transwell chamber. Then, the chamber was placed in a well of a 24-well plate with 750 μL serum-containing medium per well. Cells were cultured for 16-24 h at 37°C, in 5% CO2, fixed and were stained with crystal violet 0.05%.

### Invasion assay

Invasion assay was performed using Matrigel invasion chambers (Corning, Cat#354483). Chambers were rehydrated according to manufacturer's protocol. Similar to migration assay, cells were starved for 5-6 hrs, then trypsinized and seeded in transwell chambers containing serum-free medium. Chambers were placed in a well with serum-containing medium. Cells were cultured for 16-24 h at 37°C, in 5% CO2, fixed and were stained with crystal violet 0.05%.

### Xenograft studies

Stable pools of MDA-MB-231 and MDA-MB-435s cells expressing full length human MALAT1 or the empty vector were injected (2×10^6^ cells/site in 200μl PBS) subcutaneously into each rear flank of NOD-SCID mice (8 weeks old) using a 26 gauge needle. Tumor growth was measured bi-weekly. Tumor volume was calculated using the formula, Tumor Volume= (Length X Width2)/2. MDA-MB-435s cells are GFP labeled, so their metastatic potential can be examined by looking at the expression of GFP in organs distant from the site of injection. In the present study, after 6 weeks, the lungs were removed and metastases were visualized using GFP expression.

Animals were sacrificed by a lethal dose of anesthesia. Mice were held in specific pathogen free conditions. All animal experiments were performed in accordance with the guidelines of the Hebrew University committee for the use of animals for research.

### Survival analysis

Prognostic significance of MALAT1 in predicting Disease Specific Survival (DSS) in breast cancer patients was examined in the Curtis et al., microarray data set (n=1992) [91]. Univariate and multivariable analyses were performed using log-rank test and Cox regression model, respectively using MedCalc (MedCalc Software, Ostend, Belgium). Survival plots were created by using Kaplan-Meier methods using GraphPad Prism software (GraphPad Software, La Jolla, CA).

### Correlation analysis

The expression data of 250 cancer-related genes (459 hits including hits from EMT-associated genes and transcription factors) were retrieved from Curtis clinical dataset [91]. Pearson Correlation Coefficient (PCC) was calculated as a measure for estimating the correlation between the expression of MALAT1 and each of the genes of interest in patients from different subtypes.

## SUPPLEMENTARY FIGURES AND TABLES








