# Supplementary material for: Functional and prognostic significance of long non-coding RNA MALAT1 as a metastasis driver in ER negative lymph node negative breast cancer
Source: Oncotarget. 2016 May 26;7(26):40418–36. doi: 10.18632/oncotarget.9622 (PMC5130017; doi:10.18632/oncotarget.9622)
Supplement: Supplementary file 1 [file oncotarget-07-40418-s001.pdf]

SUPPLEMENTARY FIGURES AND TABLES

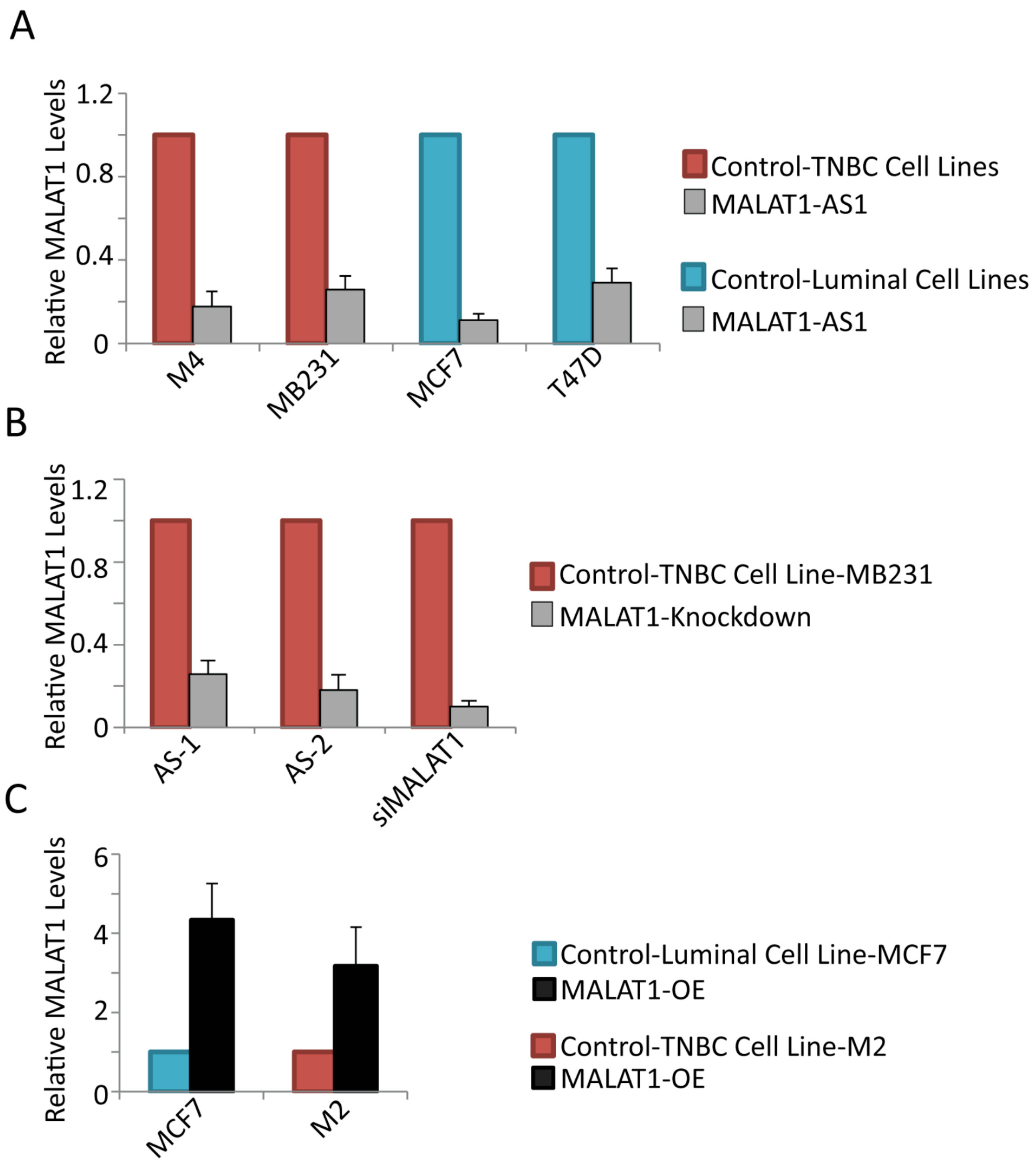

Supplementary Figure S1: MALAT1 knockdown efficiency upon A. knockdown using AS1 in BC cell lines, Scrambled AS was used as control, **B.** knockdown using AS1, AS2 and siMALAT1 in MDA-MB-231 cell line, Scrambled AS and siGL3 were used as control. **C.** hMALAT1 full length overexpression in MCF7 and M2 cell lines.

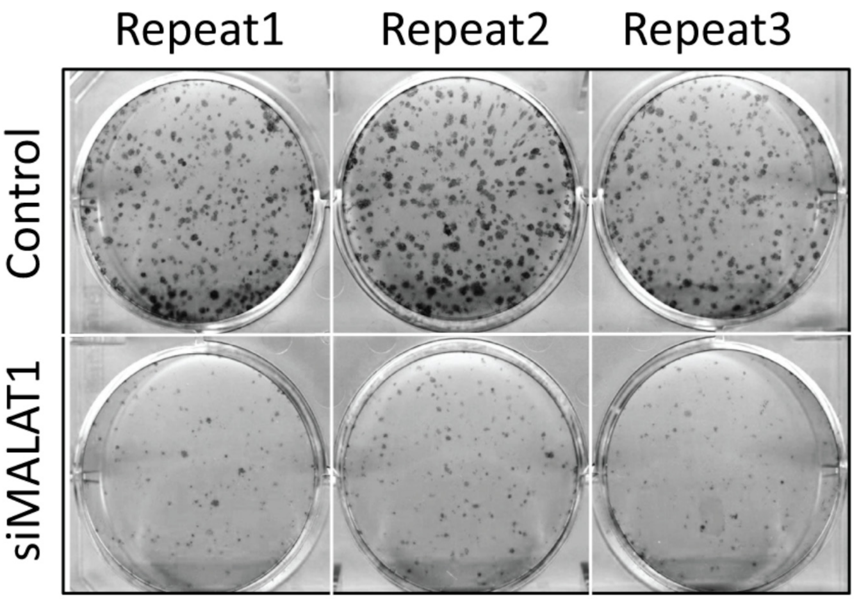

Supplementary Figure S2: Clonogenic (Plastic colony formation) assay in control and MALAT1-depleted MDA-MB-231 cells using siRNAs. GL3 siRNA was used as control.

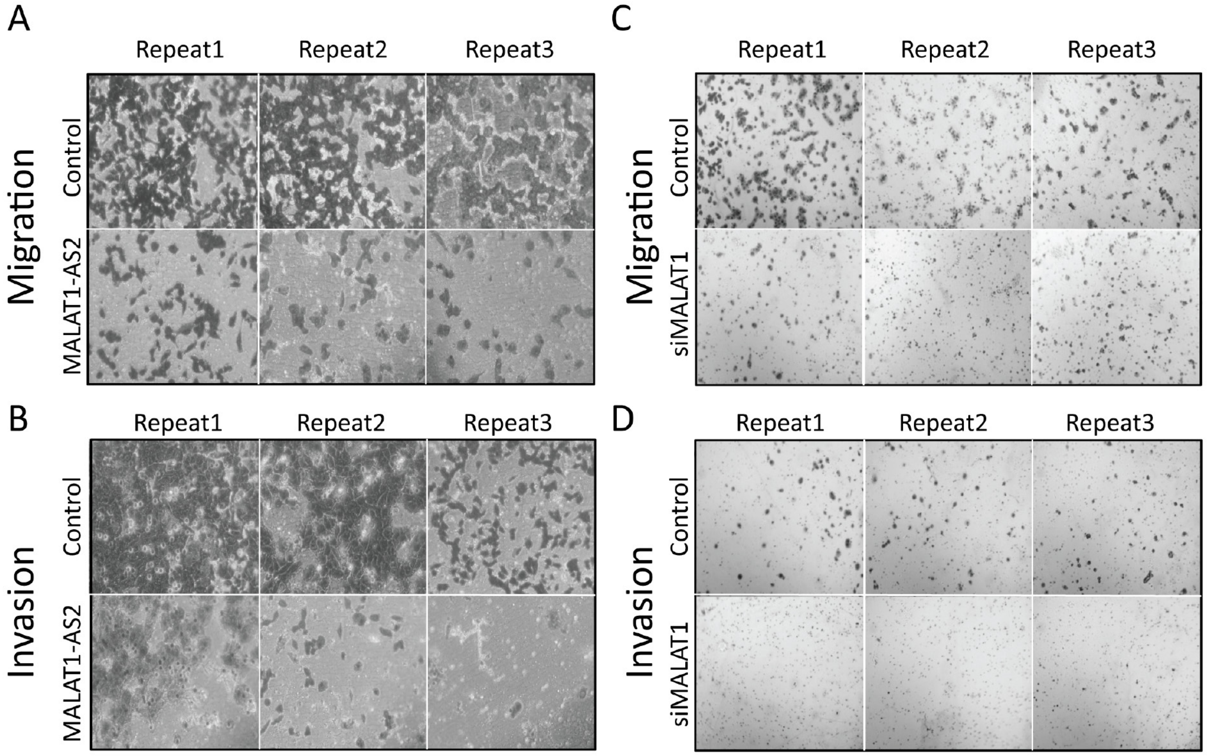

Supplementary Figure S3: Reduced levels of A. Migration and **B.** invasion upon knockdown of MALAT1 using another independent MALAT1-specific ASO (AS-2) in MDA-MB-231 cells. Reduced levels of **C.** Migration and **D.** invasion upon knockdown of MALAT1 using MALAT1-specific siRNAs in MDA-MB-231 cells.

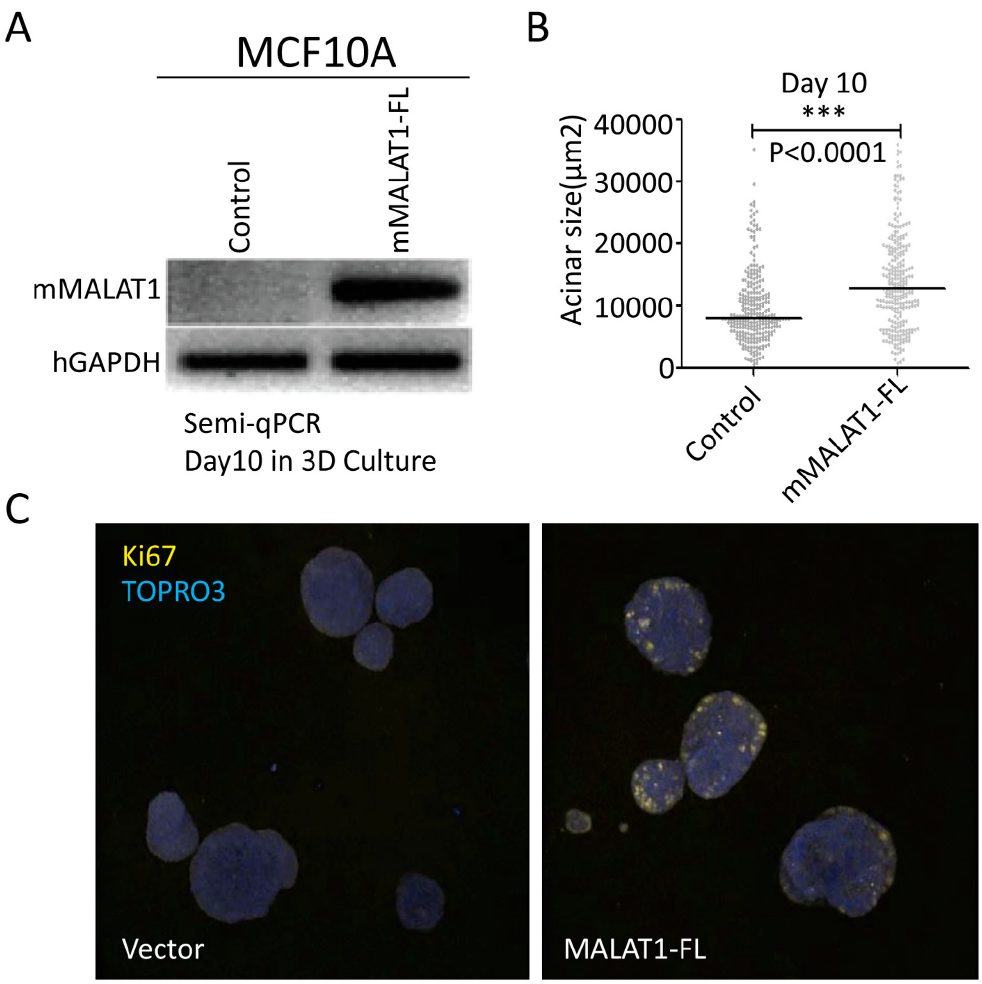

Supplementary Figure S4: MALAT1 overexpression induces hyper-proliferation in MCF10A. A. mMALAT1 overexpression in MCF10A cells. **B.** Acini Structure size in control and MALAT1-overexpressing MCF10A. **C.** Ki67 labeling in control and MALAT1-overexpressing MCF10A.

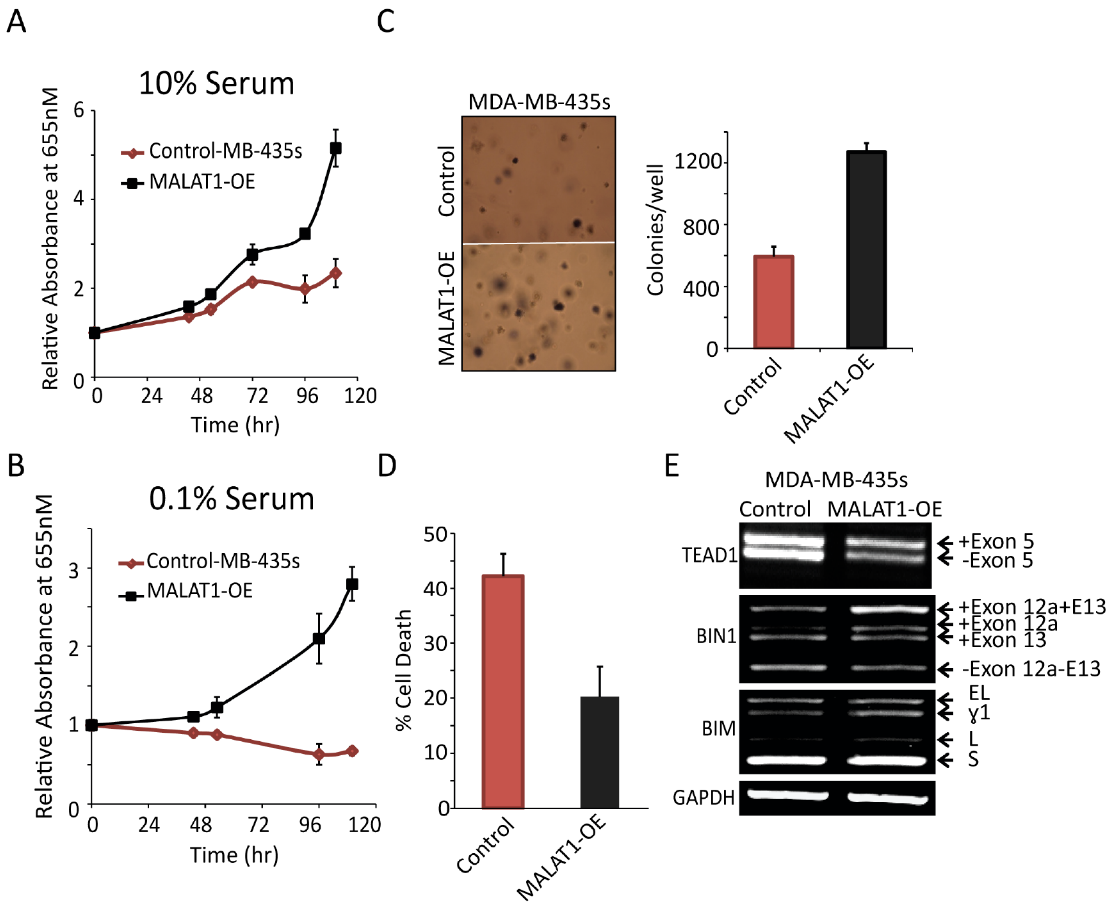

Supplementary Figure S5: MALAT1 increases the oncogenic potential of MDA-MB-435s cells A. MDA-MB-435s cells were transduced with lenti viruses encoding an empty vector or full length human MALAT1. These cells were seeded on 96 well plates and proliferation was measured as described in materials and methods section. The error bars represents SD from 6 repeats. **B.** MALAT1 enhances proliferation of breast cancer cells. Cells described in (A) were seeded on 96 well plates and proliferation was measured under 0.1% serum condition. MALAT1 over expressing cells showed increased proliferative capacity even under starvation condition. The error bars represents SD from 6 repeats. **C.** Cells described in (A) were seeded into soft agar and colonies were counted after 28 days. Graph represents the average and SD of number of colonies/well (N=3). Cells over expressing MALAT1 formed larger number of colonies in soft agar compared to empty vector. **D.** Trypan blue exclusion assay of cells described in (A) to 0.1% serum and 1μM anisomycin for 24 hours. MALAT1 overexpression significantly inhibited anisomycin-induced cell death. **E.** MALAT1 overexpression affects alternative splicing of SRSF1 targets. We found an increase in the inclusion of exon 5 of *TEAD1* in cells overexpressing MALAT1, similar to the effect of SRSF1 overexpression. We found increase in the anti-apoptotic isoforms containing exon 12A of the *BIN1* gene and isoform g1 of the apoptotic gene *BIM* in MALAT1-overexpressed cells.

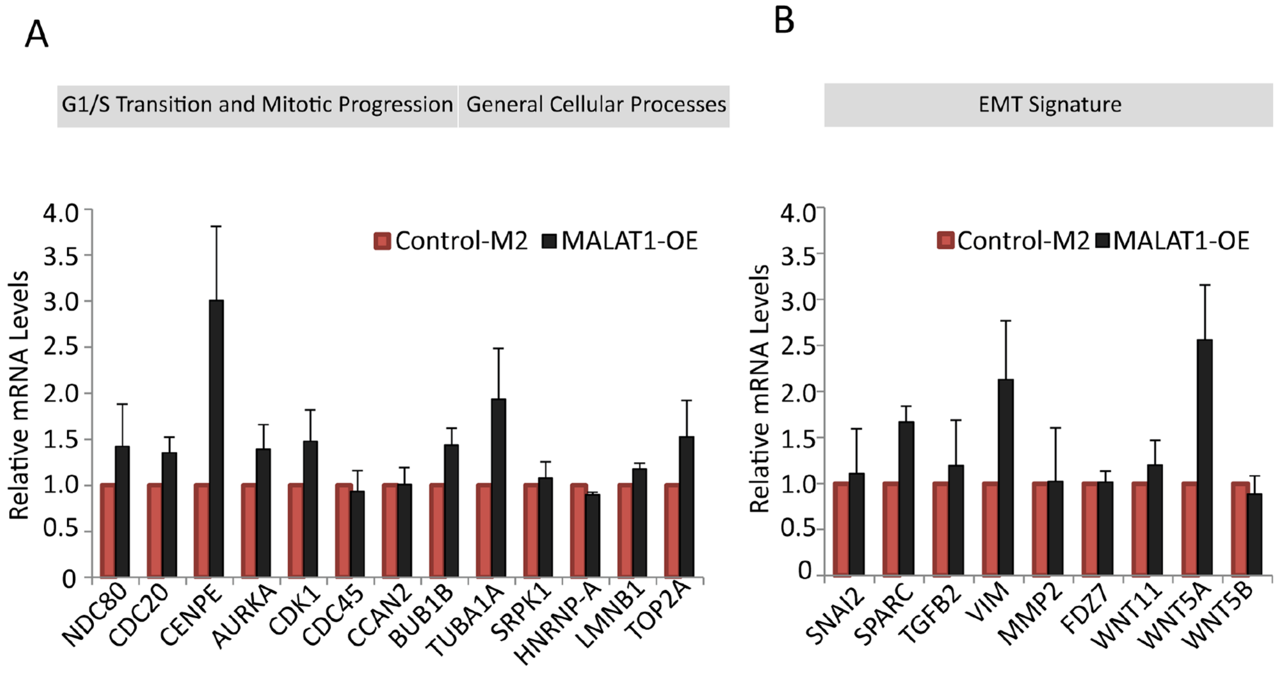

Supplementary Figure S6: MALAT1 regulates the expression of genes involved in cell cycle and EMT. RT-qPCR analyses to detect relative levels of mRNA of genes that are involved in cell cycle **A.** and EMT **B.** in control and MALAT-overexpressed M2 cells.

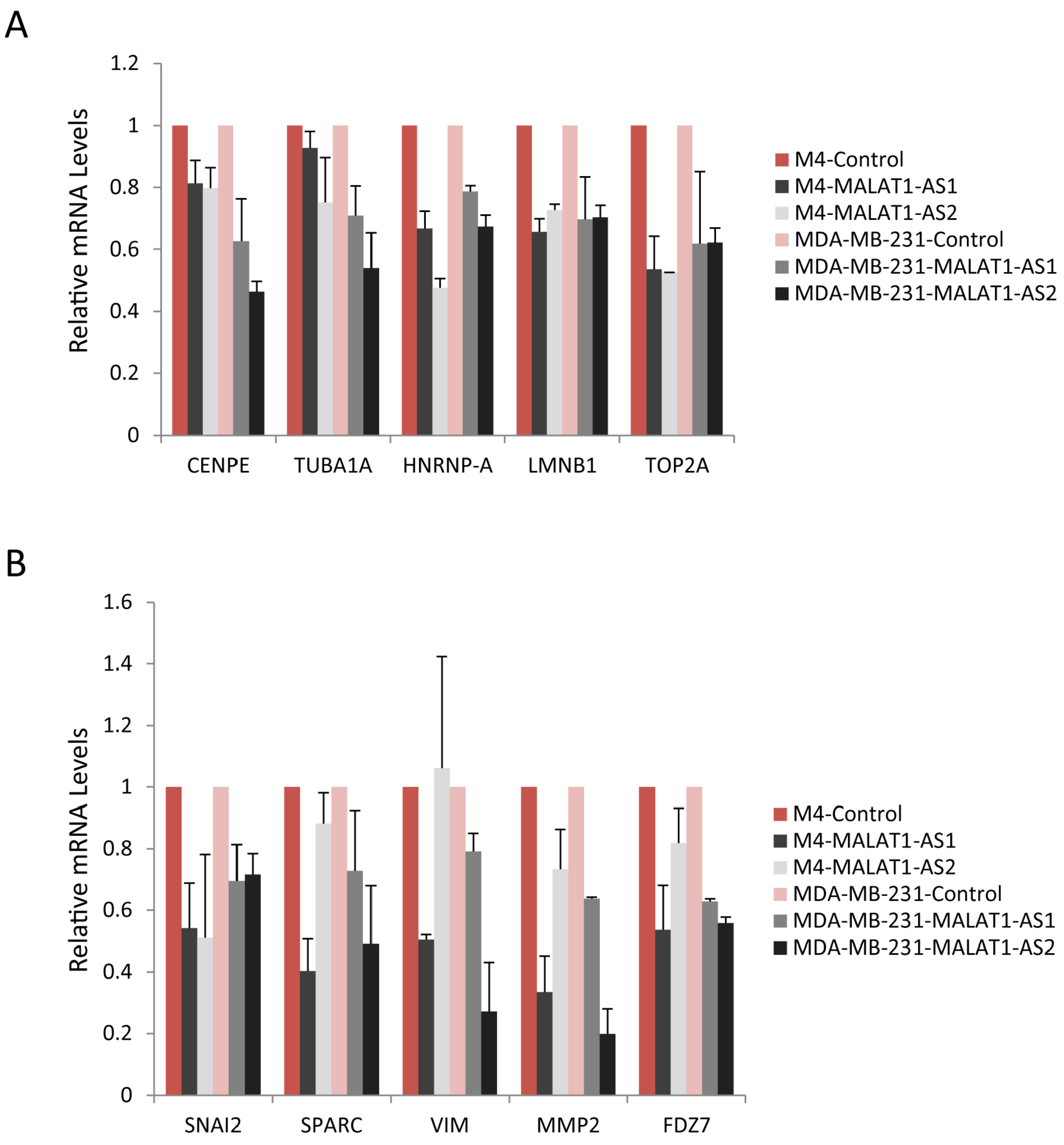

Supplementary Figure S7: MALAT1 regulates the expression of genes involved in cell cycle and EMT. RT-qPCR analyses to detect relative levels of mRNA of genes that are involved in cell cycle **A.** and EMT **B.** in control and MALAT-depleted cells (M4 and MDA-MB-231) using two independent MALAT1-specific ASOs (AS-1 and AS-2).

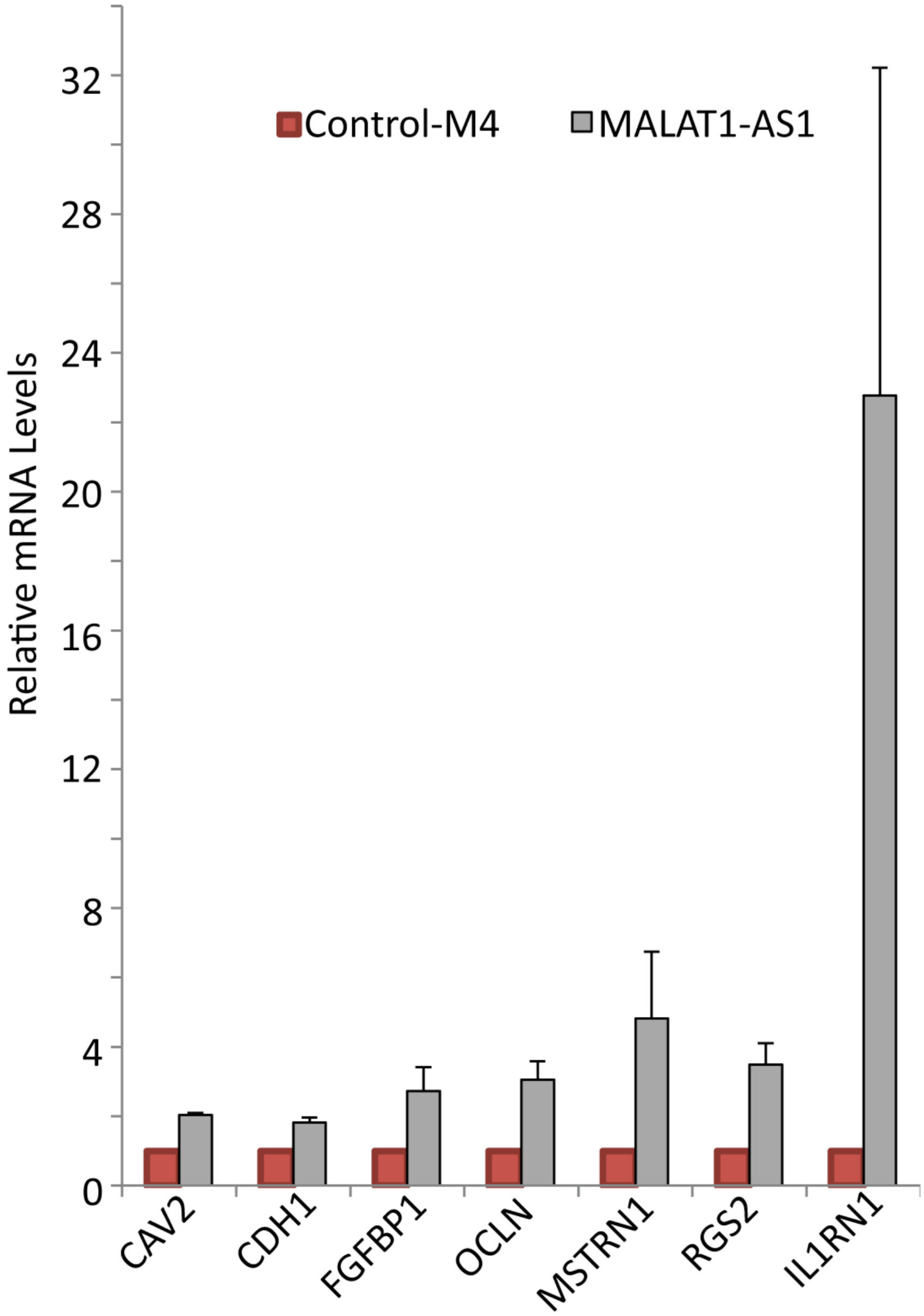

Supplementary Figure S8: MALAT1 regulates the expression of genes involved in EMT. RT-qPCR analyses to detect relative levels of mRNA of genes that are involved in EMT in control and MALAT-depleted M4 cells.

Supplementary Table S1: EMT profiler assay in control and MALAT1-depleted M4 cells.

See Supplementary File 1

Supplementary Table S2: Expression correlation analysis.

See Supplementary File 2

Supplementary Table S3: Primer list.

See Supplementary File 3
